# A Process Analytical Concept for In-Line FTIR Monitoring of Polysiloxane Formation

**DOI:** 10.3390/polym12112473

**Published:** 2020-10-25

**Authors:** Julia C. Steinbach, Markus Schneider, Otto Hauler, Günter Lorenz, Karsten Rebner, Andreas Kandelbauer

**Affiliations:** 1 School of Applied Chemistry, Reutlingen University, 72762 Reutlingen, Germany; Julia.Steinbach@Reutlingen-University.DE (J.C.S.); markus.schneider@reutlingen-university.de (M.S.); guenter.lorenz@reutlingen-university.de (G.L.); karsten.rebner@reutlingen-university.de (K.R.); 2 Reutlingen Research Institute, 72762 Reutlingen, Germany; otto.hauler@reutlingen-university.de

**Keywords:** polysiloxane, process analysis and process control, FTIR spectroscopy, multivariate data analysis, batch modelling, reaction trajectories

## Abstract

The chemical synthesis of polysiloxanes from monomeric starting materials involves a series of hydrolysis, condensation and modification reactions with complex monomeric and oligomeric reaction mixtures. Real-time monitoring and precise process control of the synthesis process is of great importance to ensure reproducible intermediates and products and can readily be performed by optical spectroscopy. In chemical reactions involving rapid and simultaneous functional group transformations and complex reaction mixtures, however, the spectroscopic signals are often ambiguous due to overlapping bands, shifting peaks and changing baselines. The univariate analysis of individual absorbance signals is hence often only of limited use. In contrast, batch modelling based on the multivariate analysis of the time course of principal components (PCs) derived from the reaction spectra provides a more efficient tool for real-time monitoring. In batch modelling, not only single absorbance bands are used but information over a broad range of wavelengths is extracted from the evolving spectral fingerprints and used for analysis. Thereby, process control can be based on numerous chemical and morphological changes taking place during synthesis. “Bad” (or abnormal) batches can quickly be distinguished from “normal” ones by comparing the respective reaction trajectories in real time. In this work, FTIR spectroscopy was combined with multivariate data analysis for the in-line process characterization and batch modelling of polysiloxane formation. The synthesis was conducted under different starting conditions using various reactant concentrations. The complex spectral information was evaluated using chemometrics (principal component analysis, PCA). Specific spectral features at different stages of the reaction were assigned to the corresponding reaction steps. Reaction trajectories were derived based on batch modelling using a wide range of wavelengths. Subsequently, complexity was reduced again to the most relevant absorbance signals in order to derive a concept for a low-cost process spectroscopic set-up which could be used for real-time process monitoring and reaction control.

## 1. Introduction

The stable polymer backbone of alternating silicon and oxygen atoms and the absence of carbon–carbon bonds is the basis of various beneficial properties of polysiloxanes, like UV-resistance, thermal stability and longevity [1,2,3,4,5]. They find numerous applications ranging from pressure sensitive adhesives [6] over thermally resistant coatings [7] to encapsulation of light emitting diodes [8,9]. A variety of different products can be produced on the basis of one and the same general reaction by varying the reaction conditions. The synthesis of polysiloxanes is dominated by hydrolysis and condensation reactions of monomeric and oligomeric educts, intermediates and products. Hydrolysis and condensation occur simultaneously and are in equilibrium with each other. Small changes in the reaction conditions have a strong influence on the composition of the reaction mixture, the course of the further reaction and thus the reaction products obtained and their purity [10,11,12]. In-line real-time information about the process state and the intermediates is required to achieve an improved process understanding, reproducibility and assured product quality. Additionally, in-depth process knowledge and powerful, affordable process-control tools are also the basis for targeted product design, process intensification and eventually the transition to continuous production in the future.

Many measurement techniques have been used in the past to follow the reaction course during polymerization, such as calorimetric, chromatographic, ultrasonic, dielectric or various spectroscopic methods [13]. Optical spectroscopy plays an especially important role in this context since, in contrast to most other methods, it offers the potential of in-situ measurements with rapid generation of relevant information. Optical spectroscopy allows real-time, multivariate and non-destructive analysis of complex reaction mixtures and is a valuable in-line process analytical technology [13,14]. FTIR spectroscopy in particular is of great interest due to the chemical information directly accessible and its high specificity in the fingerprint region (<1500 cm^−1^) [15,16]. It has already proven useful for the characterization of sol-gel based processes [17,18,19]. Attenuated total reflection probes (ATR) allow in-line measurements even in demanding measurement environments and reaction mixtures with highly oxidative and corrosive substances, such as chlorosilanes or strong acids and bases. The low susceptibility to disturbances by light scattering effects caused by bubbles or particles makes ATR a suitable measurement principle for use in heterogenous reactions [20,21].

However, high-resolution FTIR spectrometers covering the full range of wavelengths are relatively expensive which limits their attractiveness as in-line process analyzers. Cost-reduction can be achieved by designing a specialized spectrometer “around the required wavelengths” by omitting all unnecessary components. Such low-cost spectrometers proved already to be potentially suitable for analysis of chemical compositions by means of different measuring techniques [22,23]. However, typically they provide lower signal-to-noise ratio and resolution [23,24]. This downside is frequently overcome by the benefit provided by quick real-time information and can partly be compensated by multivariate data analysis (MVA). MVA uses all physical and chemical information contained in the spectroscopic signals and allows to identify and extract the portion of information useful for controlling the process. It reduces data complexity and enables the large data volume from multivariate measurements to be rapidly processed allowing for real-time monitoring [25]. Deconvolution of mixture spectra allows the separation of noise and superimposed signals. Suitable wavelength ranges for efficient process monitoring can be systematically identified and subsequently be exploited by low-cost spectrometers.

Here, the potential of in-line ATR FTIR spectroscopy as a tool for a low-cost, in-situ real-time process analyzer for monitoring polysiloxane synthesis is investigated. In a first step, the preparation process is followed by in-line ATR FTIR spectroscopy using an immersion probe. The evolving reaction mixture is analyzed by assigning the observed absorption bands to functional group vibrations. In a second step the so-called batch modelling is performed, i.e., the chemometric reduction of the reaction spectra to a few underlying (mathematical) principal components is performed via PCA. The influence of different reaction conditions is investigated by comparing different reaction trajectories based on these principal components. Abnormal reaction conditions lead to deviating product quality. It is shown that reaction profiles deviating from the standard conditions can be recognized early on as reaction trajectories diverging from the standard reaction course. Finally, it is evaluated to what extent a low-cost in-line spectrometer with highly specific components for the problem at hand could be suitable for real-time reaction tracking and process control by further reducing the spectral information to a few characteristic wavelengths. To mimic such a low-cost spectrometer, the batch model is recalculated with a reduced data set and its validity is tested.

## 2. Materials and Methods 

### 2.1. Chemicals

Trimethylchlorosilane (TMCS, CAS 75-77-4) was purchased from Thermo Fisher GmbH (Kandel, Germany). Sodium silicate (CAS 1344-09-8) was purchased from Carl Roth GmbH + Co. KG (Karlsruhe, Germany) as an aqueous solution with 28.5% SiO_2_ and 7.8% Na_2_O. Concentrated hydrochloric acid (HCl, CAS 7647-01-0), isopropyl alcohol (IPA, CAS 67-63-0) and a 99% high purity isomer mixture of xylene (CAS 1330-20-7) were obtained from Grüssing GmbH (Filsum, Germany).

### 2.2. Polysiloxane Synthesis

**Figure 1:**
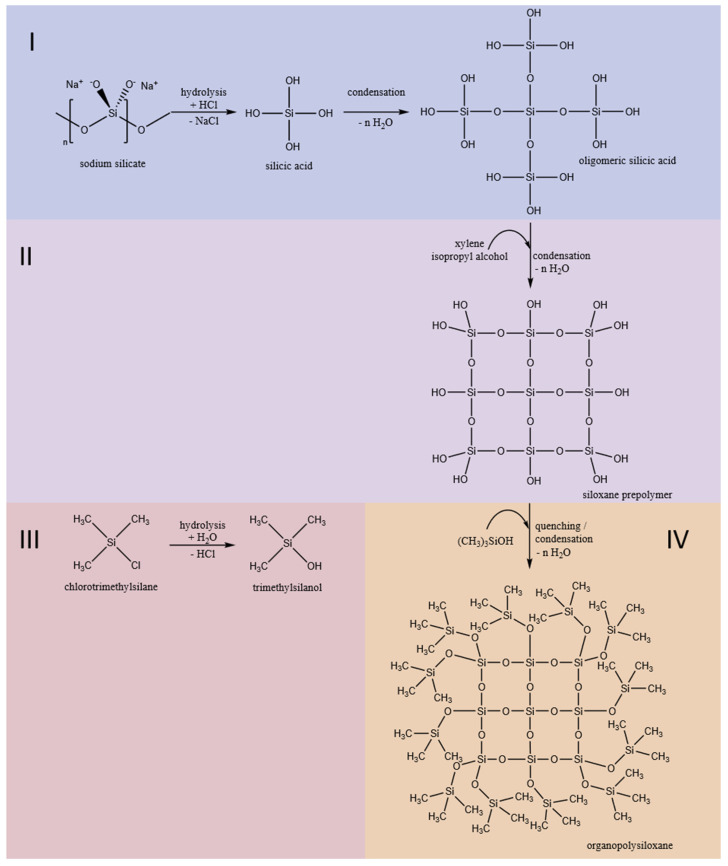
Schematic diagram for the synthesis of MQ polysiloxane. The colors displayed indicate the reaction steps as analyzed in the batch modelling analysis: (**I**) Hydrolysis of sodium silicate to silicic acid and subsequent condensation to oligomeric silicic acid, (**II**) addition of the organic phase (IPA/xylene) and further condensation and formation of a siloxane prepolymer, (**III**) hydrolysis of the M building block TMCS yielding trimethylsilanol and (**IV**) condensation to the siloxane pre-polymer, forming the MQ polysiloxane.

The siloxane considered in this work is a simple MQ polysiloxane, consisting of a monofunctional M unit (TMCS) and a tetra functional Q unit (sodium silicate precursor) [2]. Three syntheses were conducted based on the procedure given in [26] under variation of the overall reactant concentrations and, consequently at varying levels of HCl catalyst. The final concentrations of HCl employed were 4.6 mol∙L^−1^ (C4.6), 3.7 mol∙L^−1^ (C3.7) and 2.2 mol∙L^−1^ (C2.2). Concentrated HCl was added to a three-necked round bottom flask, diluted to the required acid concentration and cooled below 10 °C; 32.8 g of the precursor sodium silicate (Q unit) was diluted with 32.8 g H_2_O. This mixture was added dropwise over 3 min to the acidic reaction medium under vigorous stirring. A mixture of IPA mixed with xylene (37.3 g/13.9 g IPA/xylene) was then added dropwise yielding an organic phase. To prevent further condensation, TMCS (M unit, M:Q = 42.9 wt% TMCS) was added dropwise over 5 min as a quenching agent for end-capping. After complete addition of the TMCS, the mixture was slowly heated to 70 °C and kept under reflux for 2 h. The synthesis is shown schematically in Figure 1.

### 2.3. Process Analytical Monitoring and Data Analysis

For in-line reaction monitoring of polysiloxane synthesis, an ATR FTIR probe with a diamond crystal as internal reflectance element, (DiComp, Mettler Toledo, Germany) coupled with React IR 45 m spectrometer (Mettler Toledo, Gießen, Germany) was used. The spectra were acquired in a range from 1800 to 650 cm^−1^, with a resolution of 4 cm^−1^ and a measurement interval of 30 s per spectrum. For all three syntheses a total of 550 spectra were recorded. Chemically significant and assignable vibrations occurred in a range from 1300 to 800 cm^−1^. Accordingly, this range was used for the spectral analysis.

For multivariate data analysis and data processing the software Unscrambler X (10.5, CAMO Analytics AS, Oslo, Norway) was used. All spectra were baseline corrected, and processed using Principal Component Analysis (PCA) for data reduction and investigation of superimposed signals. Models based on two principal components explained the data variance sufficiently well and were selected for the batch modelling. The models were verified by full cross validation.

PCA is a modelling technique, which allows a reduction of the data dimension with the smallest possible loss of information. The data, consisting of n objects and p variables, is projected into a new coordinate system with the center axis pointing towards the direction of maximum variance. The axes of the new coordinate system are called Principal Components (PCs). Higher PCs lie orthogonally to the previous PCs, pointing in the direction of the next largest variance in the dataset. The original data are represented by scores and loadings. Loadings provide a “weighted” relationship between the original p variables and the PC direction. The higher the loading value of the original variable, the more the main axis (PC-1) points to the direction of these original variables. Scores can be seen as coordinates of each of the n objects in the new coordinate system. The main characteristics of an object are described by the score, relative to the variables with high loadings of the same PC. The best approximation of the original data is sought by minimizing the squared distances from all n objects in the data matrix. The resulting subspace for any number of dimensions (corresponding to the number of components) provides the most accurate representation of the original data [25,27,28].

## 3. Results and Discussion

### 3.1. IR Analysis of Polysiloxane

**Figure 2:**
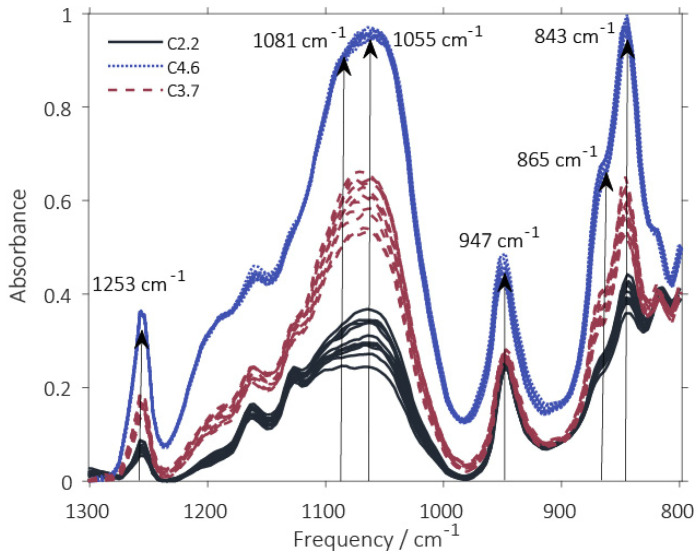
Spectra at the end of the synthesis (baseline correction, range of 1300–800 cm^−1^). Differences in absorbance and peak ratios can be seen. Arrows indicate positions of prominent frequencies. Spectra displayed black are associated with C2.2, dashed red with C3.7 and dotted blue with C4.6.

**Figure 3:**
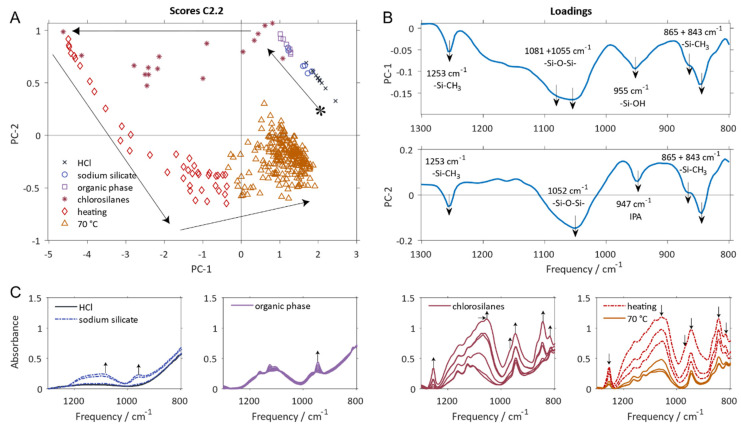
PCA model of all three syntheses (baseline correction 1800–650 cm^−1^). (**A**) Score values exemplarily for C2.2; * starting point of preparation process; arrows display the time course of the synthesis; (**B**) loading plots; (**C**) representative spectra of individual reaction steps (baseline correction 1300–800 cm^−1^). Displayed colors refer to reaction steps as defined in Figure 1.

Synthesis of polysiloxane was performed at three different reactant concentrations and, consequently at three different levels of acid catalyst in order to evaluate the potential of ATR FTIR spectroscopy to track differences in the reaction course under varying process conditions. Polysiloxane synthesis was monitored in-line using ATR FTIR spectroscopy. Figure 2 shows the infrared spectra of the reaction mixtures for the three syntheses at the end of the reaction. The infrared spectra obtained from the reaction medium during the various individual stages of polysiloxane preparation as defined in Figure 1 are shown in Figure 3C.

The peak assignments are summarized in Table 1. The sharp peak at 1253 cm^−1^ was assigned to -Si(CH_3_)_3_ groups [29]. Si-CH_3_ group vibrations were assigned to the absorbance signals at 865 cm^−1^ and 843 cm^−1^. The broad peak around 1080–1050 cm^−1^ was attributed to the asymmetric stretching -Si–O–Si– vibration with different polymerization degrees. 

An increase in absorbance of the vibrations occurring at higher wavenumbers and, in turn, at higher excitation energy, is correlated to a higher crosslinking degree of the –Si–O–Si– network [30,31,32,33]. Hence, the observed shifts in absorbance towards higher wavenumbers are attributed to highly crosslinked –Si–O–Si– groups (here Q building block, see Figure 1, step 4) in the skeletal structure within the sol. An increase in absorbance at higher vibrational frequencies thus also indicates an increased size of silicic acid oligomers and siloxane pre-polymers. 

Vibration intensity of surface-bound –Si–O–Si– groups (~1050 cm^−1^) decreases with increasing siloxane pre-polymer size [34]. Silanol groups from silicic acid or surface bound -Si-OH groups are found at ~950–960 cm^−1^, as broad -Si-OH stretching vibration [35]. The characteristic sharp absorbance band of the –C–O vibration from IPA around 950 cm^−1^ [36,37] (here 947 cm^−1^) is superimposed by the absorption band of the –Si–OH vibration. Therefore, a separate quantitative evaluation is not directly possible. The overall band intensities vary with varying concentrations of the reactant, while the spectral patterns remain similar. With the C4.6 polysiloxane the absorbance of all bands was highest since the least amount of water was present in the reaction mixture and the concentrations of all components was highest. Overall band intensities drop with decreasing concentrations in the order C4.6 > C3.7 > C2.2. While the main bands can be assigned more or less correctly and the spectra can be qualitatively discussed, careful inspection of the reaction spectra shows that the peaks are strongly overlapping and peak shifts occur as the reaction progresses. Hence, a quantitative evaluation and tracking of the reaction progress using a univariate approach is not straight forward. Simultaneous hydrolysis and condensation of –Si–O–Si– bonds during synthesis influence the same wavelengths. Using reaction profiles based on single wavelengths, it is not possible to correctly evaluate the information carried by peak shifts, such as the shift of the –Si–O–Si– vibration towards higher frequencies with increasing siloxane pre-polymer size and higher degree of condensation. Therefore, in the next step, MVA was applied to identify spectral patterns, reduce complexity of the spectroscopic dataset and apply batch modelling based on principal components. 

### 3.2. Batch Modelling of the Polysiloxane Preparation Process using PCA

In batch modelling, independent information hidden in the time-dependent spectral data is extracted as mathematical entities called principal components (PCs). Plotting PCs against each other, reaction trajectories in the principal component space are obtained which depict the characteristic changes in orthogonal information over time during chemical synthesis. Figure 3A shows the scores plot for reaction C2.2. Figure 3B shows the corresponding loading plots of the PCA for all syntheses. For this PCA, all absorbance values from 1300 to 800 cm^−1^ for the complete reaction time profiles of all three reactions were used. The explained variances are 83.4% and 11.8% for PC-1 and PC-2, respectively (see Table 2). With an overall explained variance > 95.0%, the in-line spectroscopic data can be described sufficiently well using only two PCs. This means that only two independent (orthogonal) underlying variables are sufficient to pinpoint the characteristic changes taking place during polysiloxane preparation. These changes follow a characteristic pattern in the PCA space which can be assigned to the individual phases of the reaction as indicated by the color code used in Figure 1 and Figure 3A. 

In batch modelling, ideally the complete preparation process is envisaged starting from the charging of the reactants and covering all process steps until completion of the synthesis. Since many faulty batches can be traced back to simple errors during dosing and mixing of the reagents, this is useful from an industrial point of view. Hence, in our study the complete time course of process spectra starting with the charging of hydrochloric acid has been considered and was used for the analysis.

The starting point of the measurements is indicated by an asterisk in Figure 3A. At this stage, hydrochloric acid was added to the reaction vessel. Subsequent sodium silicate addition is indicated by blue open circles. The time course of the reaction is indicated by the arrows in Figure 3A. The individual symbols depicted in the score plot and the applied color code follows the individual reaction steps as defined in Figure 1.

With the addition of sodium silicate, the PC-1 score values decrease while PC-2 scores increase. The addition of organic phase (xylene/IPA) results in a slight initial increase on PC-1 scores, but the overall trend continues. A massive decrease in PC-1 scores is visible for the addition of chlorosilanes, while, at this stage, PC-2 remains nearly constant. This is the largest information contribution of the first PC. The largest contribution of PC-2 occurs during heating, whereby the score values decrease. With progress in heating, the score values of PC-1 increase. After reaching the target temperature of 70 °C the scores show fluctuations on PC-1 and PC-2 around a respective mean value.

The loading plots for the two principal components and the most prominent changes in wavenumbers in the corresponding original spectra are given in Figure 3B. The loadings plot informs on the characteristic information which is extracted from the original spectra and whose time-dependent changes are summarized in the scores plot for the individual samples. PC-1 loadings (Figure 3B) are mainly negative. This correlates with a general increase in absorbance at these wavelengths for all measurements with negative score values. The wavelengths providing the most information are 1253 cm^−1^_,_ 865 cm^−1^ and 843 cm^−1^_,_ which can be correlated with –Si–CH_3_ stretching vibrations, 1081 cm^−1^ and 1055 cm^−1^ which are assigned to -Si-O-Si- asymmetric stretching vibrations of different condensation states [30] and 955 cm^−1^ which is associated with the stretching vibration of -–Si–OH groups (Table 1). In PC-2, similar wave numbers are predominant as in PC-1, but the overall intensity is smaller. The stretching band of Si–OH around 955 cm^−1^ is narrower than on PC-1 and shifted towards 947 cm^−1^. This indicates a decrease in the amount of Si-OH as the PC-2 score value decreases. The asymmetric stretch vibration –Si–O–Si– at 1081 cm^−1^ is not present in PC-2. 

The synthesis is based on a sol-gel process characterized by hydrolysis of –Si–O–Si– bonds and condensation of -Si-OH groups, which is followed by end-capping by organosilanols. Consequently, the vibration signals of these groups dominate the loadings of the PCA (compare Figure 2 and Figure 3). The combined consideration of the information of loadings and scores makes it possible to detect and visualize chemical or physical changes in the analyzed reaction mixture. Here, the dominating effects can be identified as the hydrolysis and condensation reactions of different functional groups. The pattern of the scores allows to follow the course of the reaction over time.

The addition of sodium silicate to HCl (single spectra shown in Figure 3C), leads to an increase of intensity at 1300–950 cm^−1^, in particular at 1081 cm^−1^ and 1055 cm^−1^ on PC-1, which is assigned to –Si–O–Si– asymmetric stretching vibrations. Simultaneously, the intensity of the -Si-OH stretching vibrations at 955 cm^−1^ increases. This reflects the hydrolysis of silica, which occurs together with the decrease of intensity at 1081 cm^−1^ which is indicative for cleavage of -Si-O-Si- bonds of higher polymerization degrees.

The addition of the organic phase mainly has a diluting effect and the intensity around 947 cm^−1^ (which is assignable to the C-O stretching vibration of IPA) increases. It is noticeable, that the IPA peak superimposes the broader -Si-OH stretching vibration (Figure 3C) and can be assigned to an increase of PC-2 and the corresponding loading. The hydrolyzation of sodium silicate continues as indicated by the decrease of PC-1 score values, which is correlated to an increase in intensity of the -Si-OH stretching vibration.

The addition of chlorosilanes massively increases overall intensity (decrease of PC-1) and introduces -Si-CH_3_ vibrations (1253 cm^−1^, 865 cm^−1^ and 843 cm^−1^) to the loadings. The intensity of -Si-OH stretching vibration (PC-1, 955 cm^−1^) increases due to hydrolysis of chlorosilanes to organosilanols. Concurrently, the in-situ generated silicic acid condenses and builds oligomeric silicic acid with lower polymerization degrees, leading to increasing intensities of the –Si–O–Si– asymmetric stretching vibrations at 1055 cm^−1^ and slight decrease of PC-2 score values.

With heating up to 70 °C, a decrease on PC-2 scores is observable, which can be correlated with an overall decrease in spectral intensity, and a shift for the -Si-O-Si- peak to 1052 cm^−1^ (see Figure 3B). The relative intensity of -Si-CH_3_ vibrations, especially at 1253 cm^−1^ and 845 cm^−1^, can be correlated with the functionalization of oligomeric silicic acid with lower polymerization degree. This becomes evident through the predominance of the lower -Si-CH_3_ frequency (at 865 cm^−1^, the relative intensity decreases). Moreover, the -Si-OH peak disappears and the -C-O stretching vibration of IPA at 947 cm^−1^ remains. This indicates the formation of higher condensed oligomeric silicic acid and siloxane pre-polymer (see Figure 1) by condensation (decrease of -Si-OH intensity).

PC-1 is mainly dominated by hydrolysis effects which can be concluded from an increase in -Si-OH vibrations, the shift from highly condensed -Si-O-S- vibrations from 1081 cm^−1^ to 1055 cm^−1^, which corresponds to the dissolution of sodium silicate, and the introduction of -Si-CH_3_ vibrations with the addition of organofunctional groups. PC-2 can be mainly associated with increasing -Si-O-Si- vibrations at 1052 cm^−1^ through smaller newly formed silica particles and simultaneous decrease of -Si-OH vibration intensity. The overall intensity decrease can be correlated with exceeding the solubility limits and partly precipitation or gelation with further condensation reaction.

### 3.3. Detection of Differences between Batches

**Figure 4:**
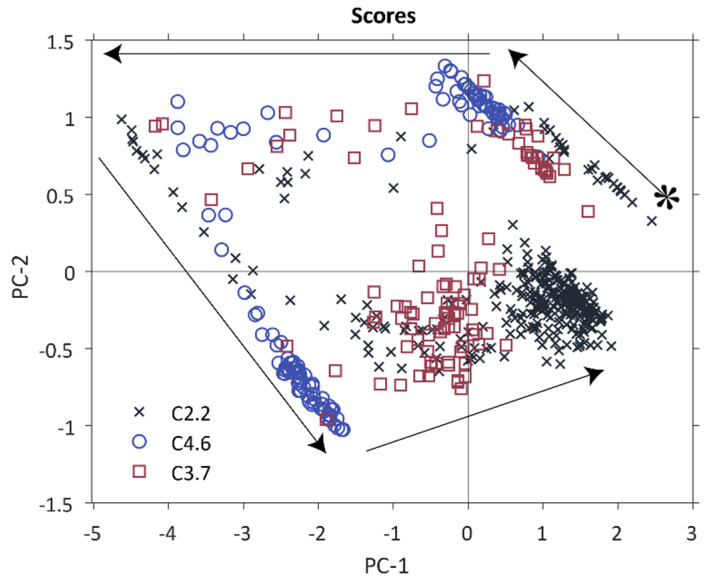
Reaction course of all three syntheses with the same PCA model as Figure 3. Only the score plot is given, for the corresponding loadings see Figure 3; * starting point of preparation process; arrows display synthesis course. Score values of C2.2 are displayed as black crosses, C3.7 as red squares and C4.6 with blue circles.

Figure 4 shows the score plot of the PCA model containing all three runs of polysiloxane preparation. The similarities in reaction course, as well as the deviations in reaction trajectories and end states are clearly visible. It is noticeable, that the reaction course of all three syntheses is similar, starting with the initial measurements of cooled aqueous hydrochloric acid in the positive PC-1 vs. PC-2 quadrant (indicated by an asterisk in Figure 4). The loadings are identical to those shown in Figure 3B as it is identical to the model shown. The diverging reaction trajectories during the heating phase for two hours at 70 °C is obvious from Figure 4. The reaction path for polysiloxane C4.6 is clearly very different from C2.2 and C3.4. Different starting points of the reaction trajectories, due to different HCl concentrations, are visible in slight deviations on PC-1 and PC-2. Batch C4.6 starts with lower score values on PC-1, and higher on PC-2, than C3.7 and C2.2. The highest starting concentration of HCl (C4.6) leads to a product with lower contribution of PC-1 and slightly lower PC-2 values. Gelation of C4.6 was observed, whereas C2.2 and C3.7 formed a turbid, emulsion of moderately high viscosity. This technological information agrees well with the interpretation of the loadings and scores presented in the previous section. The deviations in quality of the product from batch C4.6 are already visible at an early stage during synthesis in the comparatively lower values in PC-1 and PC-2 and, via the loadings plot can directly be related to an increased degree of polymerization leading to premature gelation. According to Driouich et al. (2020) [38] the gelling time decreases with an increase in HCl concentration, due to accelerated hydrolysis and the following condensation. This finding explains the occurrence of the observed early gelation already during polysiloxane preparation. Higher starting concentrations of HCl result in higher PC-2 scores, and lower PC-1 scores correlating with a higher baseline at around 1300–950 cm^−1^. This leads to the preferred formation of gels instead of sol growth [31,39] and consequently to a decrease in signal intensity through precipitation of C4.6. The products of C3.7 and C2.2 in contrast, show higher PC-2 and PC-1 scores. This indicates a lower polymerization degree and the formation of sols instead of gelation.

Hence, batch modelling allows not only to follow the reaction course but also to detect deviations from the standard reaction course at an early stage of the preparation process. Faulty batches can thereby be detected before the actual damage has occurred. Appropriate counter-measures can be initiated such as corrective measures like, for instance, addition of alkali to neutralize the excess acid or mitigating actions like discharging the faulty batch before gelation and curing takes place in the reactor and causes serious damage to the infrastructure.

### 3.4. Batch Modelling Using a Reduced Dataset and a Concept for a Low-Cost Process Spectrometer

A custom-designed low-cost spectrometer as a process analyzer is to be expected to provide lower spectral resolution and a poorer signal-to-noise ratio since only a much smaller spectral region is employed in building the multivariate calibration model. Hence, the conceptualization of a low-cost process analyzer, as an alternative to an expensive benchtop MIR spectrometer, requires a model of comparable quality despite the smaller number of wavenumbers used.

The results with full spectral range showed, as seen in Figure 3 and Figure 4, that the main spectral information is distributed over the dominant wave numbers 1081 cm^−1^, 1055 cm^−1^ and 955 cm^−1^ for hydrolysis and condensation effects. Hence, the PCA model was recalculated under use of only these wavenumbers for -Si-O-Si- and -Si-OH vibrations that have proven to be significant. The resulting model again only required two principal components for adequately describing the data set and had an explained variance of 95.0% for PC-1 and 4.7% for PC-2, given in Table 2. Most of the relevant information is contained in PC1.

**Figure 5:**
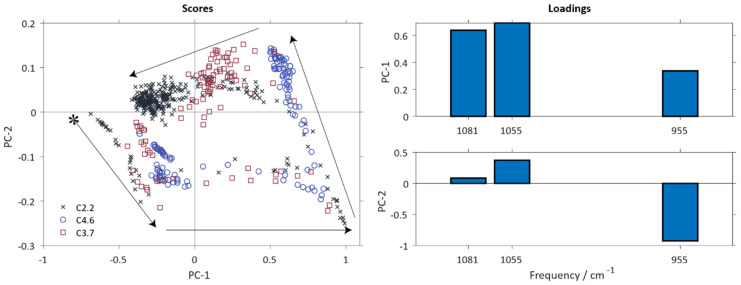
Recalculated PCA model of data matrix with significant frequencies only; * starting point of synthesis; arrows display synthesis course.

Figure 5 shows the scores and loading plots in a similar manner as given in Figure 3. Except for an inversion of the direction of the two main components, the reaction trajectories are quite similar to those shown in Figure 4. The information on the preparation process contained in the reduced model is congruent with the full model built upon the entire spectral information. PC-1 is dominated by the frequency shift of the -Si-O-Si- vibration and the increase in intensity of the -Si-OH absorbance band. It can therefore be correlated with hydrolysis reactions. PC-2 shows a decrease of the -Si-OH vibration intensity and an increase primarily of the vibration signal at 1055 cm^−1^. Thus, it displays mainly information on the condensation reactions.

The various stages of the preparation process as defined in Figure 1 are discernable from the reduced model equally well without major losses in prediction quality as are the characteristic deviations between the three reaction trajectories. The polysiloxane batch C4.6 is equally well detectable as faulty batch as it is with the full model which is evident from the scores plot given in Figure 5. As a conclusion, the three frequencies identified from principal component analysis as the ones carrying most of the relevant information on the preparation process are sufficient to allow real-time monitoring of the synthesis in a similar manner as when using the whole spectral range. This shows the potential of an inexpensive MIR spectrometer as an on-line, permanently implementable process analyzer. It is noteworthy that the three wavelengths suitable for the reduced data-based process model would probably not have been selected by a purely univariate analytical approach. Two of the three wavelengths (1055 and 1081 cm^−1^) belong to one single broad peak and the significance of the shoulder at 1081 cm^−1^ would probably have been underestimated or overlooked.

A possible setup for such a low-cost spectrometer consists of a SiC globar as a broadband light source, a MIR diffraction grating with actuator and simple photodiodes as detectors. In order to reduce the influence of heat development of the globar, an optical fiber system can be used. An Arduino can serve as data interface. For the presented application, a transmission cell can be cast from epoxy. The total costs of such a setup of would be about 5000–7000 €. This amounts to about 2–3% of the costs for a laboratory benchtop FTIR spectrometer like the one used in the present study. Another advantage of such a tailored spectrometer design is that it is relatively flexible due to the grating system. If the relevant wavelengths have been identified by preliminary studies by MVA, appropriate detectors with special sensitivity to these wavelengths can be selected and systems can be tailored as required for the application. To compensate for the lower resolution and worse signal-to-noise ratio, the MVA models will be used for analysis.

## 4. Conclusions

In the presented work it has been shown that by using inline ATR FTIR measurements of a single reaction phase and data processing by PCA the reaction course of polysiloxane preparation can be adequately followed with only two PCs. PC-1 is mainly dominated by hydrolysis effects, whereas PC-2 reflects mainly condensation and precipitation or gelation phenomena. The assignment of purely mathematical PCs was achieved by relating the relevant spectral information identified from the PCA loadings to chemical interpretation of functional group vibrations. The information extracted via PCA characterized the reaction trajectories for polysiloxane preparation. It was used for batch modelling based on PCA loadings. The main chemical information was attributed to the spectroscopic signals of the asymmetric stretch vibrations of -Si-O-Si- from compounds of different degrees of polymerization (signals at 1081 cm^−1^ and 1055 cm^−1^), and -Si-OH stretch vibrations (signal at 955 cm^−1^). The measurements allowed to distinguish between different syntheses performed with varying reactant concentrations. The combination of ATR-FTIR measurements and PCA proved to be a valuable tool for real-time reaction monitoring and identifying faulty batches.

From a second PCA model including only the most significant wavenumbers (-Si-O-Si- at 1081 cm^−1^, 1055 cm^−1^, -Si-OH at 955 cm^−1^), batch modeling yielded similar reaction trajectories as with the full dataset. With this reduced dataset, the reaction path can also be described adequately and deviations between different synthesis are shown. This results in a vast reduction of the required amount of data, and allows to aim at the implementation of a cost-efficient process analytical solution in the next step. Based on this knowledge, a concept for a low-cost process analyzer was proposed, which is intended for permanent online monitoring of the process. The described example illustrates that process analytical technology can, in principle, be implemented at reasonable costs.

## Figures and Tables

**Table 1:** Assignments of the IR bands in the spectra of the reaction mixture.

Wavenumber/cm^−1^	Assignment
1253	–Si(CH_3_)_3_
1081	–Si–O–Si– asymmetric stretching (high crosslinking degree)
1055	–Si–O–Si– asymmetric stretching (surface groups, low crosslinking degree)
~960–950	–Si–OH stretching
947	–C–O (IPA)
865, 843	–Si–CH_3_

**Table 2:** Explained variances for principal component analysis (PCA) with (a) analyzed spectral range from 1300 to 800 cm^−1^ and (b) reduced to the three most significant frequencies. Both PCA models have an explained variance >95%.

Dataset	Explained Variance PC-1	Explained Variance PC-2
1300–800 cm^−1^	83.4%	11.8%
1081, 1055, 955 cm^−1^	95.0%	4.7%
